# 98. Outcomes of Clinical Decision Support for Outpatient Management of *Clostridioides difficile* Infection

**DOI:** 10.1093/ofid/ofab466.300

**Published:** 2021-12-04

**Authors:** Tiffany Wu, Susan L Davis, Susan L Davis, Brian Church, George J Alangaden, Rachel Kenney

**Affiliations:** 1 Henry Ford Hospital, Detroit, Michigan; 2 Wayne State University, Detroit, MI; 3 Henry Ford Health System, Detroit, Michigan

## Abstract

**Background:**

Our antimicrobial stewardship program identified high rates of suboptimal metronidazole prescribing for *Clostridioides difficile* infection (CDI) within ambulatory clinics. An outpatient best practice advisory (BPA) was implemented to notify prescribers “Vancomycin or fidaxomicin are preferred over metronidazole for *C.difficile* infection” when metronidazole was prescribed to a patient with CDI.

**Methods:**

We conducted an IRB approved quasi-experiment before and after implementation of the BPA on June 3, 2020. Inclusion: Adult patients diagnosed with and treated for a first episode of symptomatic CDI at an ambulatory clinic between 11/1/2019 and 11/30/2020. Exclusion: fulminant CDI. Primary endpoint: guideline-concordant CDI therapy, defined as oral vancomycin or fidaxomicin. Oral metronidazole was considered guideline-concordant if prescribed due to cost barrier. Secondary endpoints: reasons for alternative CDI therapy, patient outcomes, prescriber response to the BPA. Descriptive and bivariate analyses were completed.

**Results:**

189 patients were included in the study, 92 before and 97 after the BPA. Median age: 59 years, 31% male, 75% Caucasian, 30% with CDI-related comorbidities, 35% with healthcare exposure, 65% with antibiotic exposure, 44% with gastric acid suppression therapy within 90 days of CDI diagnosis. The BPA was accepted 23 out of 26 times and optimized the therapy of 16 patients in six months. Guideline-concordant therapy increased after implementation of the BPA (72% vs. 91%, p=0.001) (Figure 1). Vancomycin prescribing increased and metronidazole prescribing decreased after the BPA (Figure 2). Reasons for alternative CDI therapy included medication cost, lack of insurance coverage, and non-CDI infection. There was no difference in clinical response or unplanned encounter within 14 days after treatment initiation. Fewer patients after the BPA had CDI recurrence within 14-56 days of the initial episode (27% vs. 7%, p< 0.001).

Figure 1. Guideline-concordant CDI therapy

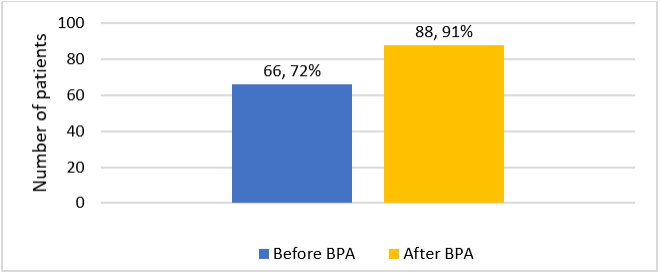

Figure 2. Specific CDI therapy

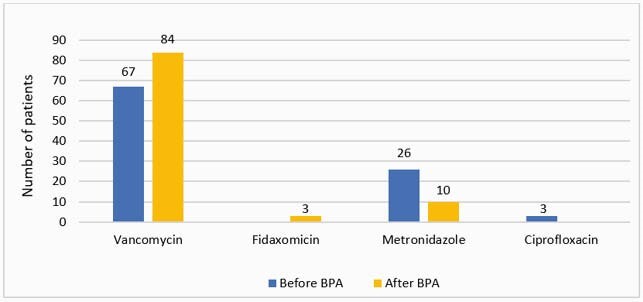

**Conclusion:**

Clinical decision support increased prescribing of guideline-concordant CDI therapy in the outpatient setting. A targeted BPA is an effective stewardship intervention and may be especially useful in settings with limited antimicrobial stewardship resources.

**Disclosures:**

**Susan L. Davis, PharmD**, Nothing to disclose **Rachel Kenney, PharmD**, **Medtronic, Inc.** (Other Financial or Material Support, spouse is an employee and shareholder)

